# Change from subcutaneous to intravenous abatacept and back in patients with rheumatoid arthritis as simulation of a vacation: a prospective phase IV, open-label trial (A-BREAK)

**DOI:** 10.1186/s13075-016-0985-2

**Published:** 2016-04-14

**Authors:** Ruediger B. Mueller, Michael Gengenbacher, Symi Richter, Jean Dudler, Burkhard Möller, Johannes von Kempis

**Affiliations:** Division of Rheumatology, Immunology and Rehabilitation, Kantonsspital St. Gallen, Rorschacherstr. 95, 9007 St. Gallen, Switzerland; Bethesdaspital, Gellertstrasse 144, 4052 Basel, Switzerland; Kantonsspital Fribourg, Chemin des Pensionnats 2, 1708 Fribourg, Switzerland; Inselspital, Sahlihaus 2, 3010 Bern, Switzerland

**Keywords:** Abatacept, Intravenous, Subcutaneous, Switch, LDA

## Abstract

**Background:**

Vacation can present a major problem to patients with rheumatoid arthritis (RA) treated with weekly subcutaneous biologics, including subcutaneous (SC) abatacept. Therefore, the replacement of four SC doses of abatacept by a single dose of intravenous (IV) abatacept may present an acceptable alternative to cover a 4-week interval needed for vacations. In the study presented, we analyzed the efficacy and safety of this intervention followed by a switch back to SC abatacept after 4 weeks.

**Method:**

This open-label, prospective, single-arm, 24-week trial recruited patients with established RA in low disease activity (LDA) or in remission on treatment with SC abatacept for at least 3 months to receive a single dose of IV abatacept (baseline) followed by a break of 4 weeks and then continuation of weekly SC abatacept from day 28 on. Disease-modifying anti-rheumatic drug (DMARD)-inadequate or biologic-inadequate responders (or both) were included.

**Results:**

The baseline characteristics of the 49 patients (per protocol) were typical for a cohort of RA patients with established disease (mean disease duration of 8.31 years) in LDA under treatment with synthetic DMARDs and a biologic. Two patients (one flare and one patient decision) dropped out of the study. The proportions of patients with disease activity score in 28 joints (DAS-28) of not more than 3.2 at day 28 were 93.9 % (95 % confidence interval (CI) 83.5–97.9) and 93.6 % (95 % CI 82.8–97.8) at the end of the study (day 168). The average DAS-28 values were 1.74 (standard deviation (SD) ± 0.72) at baseline, 2.03 (SD ± 1.03) at day 28, and 1.96 (SD ± 0.92) at the end of the study (day 168). Pre-exposure to IV abatacept and having failed methotrexate or anti-tumor necrosis factor (anti-TNF) did not influence the average DAS-28 or the proportion of patients maintaining LDA over time. The average health assessment questionnaire disability index (HAQ-DI) was stable throughout the study. Adverse events (AEs) occurred in 75 % of subjects. Four serious AEs were described during the study. None of them was related to the investigational product, and all serious AEs could be resolved during hospitalization.

**Conclusion:**

This prospective, open-label study of abatacept shows for the first time that switching from weekly SC to IV abatacept and back after 4 weeks is an effective and safe way to bridge vacations in RA patients in LDA or remission. (NCT1846975, registered April 19, 2013.)

## Background

Rheumatoid arthritis (RA) is a chronic inflammatory autoimmune disease. With the introduction of biologic disease-modifying anti-rheumatic drugs (DMARDs) more than a decade ago [1], the options for the treatment of RA have significantly improved. Biologic DMARDs (biologics) can be categorized by their different mechanisms of action and by the kind of application: subcutaneous (SC) or intravenous (IV). Abatacept is able to induce symptom reduction and to delay the progression of structural damage [2] in patients with RA. It was the first biologic to be available in both an SC [3] and an IV [4] formulation.

The efficacy and safety of IV abatacept have been well established in recent years [5]. Moreover, clinical trials comparing SC abatacept with IV abatacept have clearly demonstrated a similar efficacy [2] and safety [6] profile. A temporary withdrawal of SC abatacept for 3 months, in RA patients methotrexate-inadequate response (MTX-IR), however, resulted in increasing disease activity score in 28 joints (DAS-28) scores [7], which, in turn, decreased again after reintroduction of the drug. Switching from IV to SC abatacept appears to be associated with a persisting good efficacy of abatacept and no increase in the rate of adverse events (AEs) [8]. On the other hand, switching from SC to IV abatacept has not been investigated in a clinical trial setting so far.

Vacations may cause problems for patients with RA treated with SC biologics, because transportation of the biologics may be complicated for various reasons such as customs regulations, maintenance of the cold chain, or fragility of vials/syringes in the luggage. This is also true for SC abatacept with its weekly application schedule. In contrast to the weekly SC application, IV abatacept has to be administered only once every 4 weeks, a time period covering most vacations. Thus, to avoid issues just mentioned, patients under therapy with SC abatacept may receive a single infusion of IV abatacept before starting their vacation and will continue with SC therapy 4 weeks later once they have returned back home.

The objective of this open-label prospective study was to determine whether replacing the mandatory regular schedule of weekly SC injections of abatacept for 4 weeks by a single IV infusion is a safe and effective treatment for maintaining RA patients, who already reached a good disease status on SC abatacept, such as DAS-28-defined low disease activity (LDA) or remission.

## Methods

### Patients and study design

The A-BREAK (Abatacept Study to Omit Weekly Subcutaneous Injections in RA patients During Holiday BREAK) study was a 24-week prospective, phase IV, open-label, multi-center, single-arm clinical trial. Patients were all switched from SC (125 mg/week) to IV abatacept (dosage for body weight of less than 60 kg: 500 mg, 60–100 kg: 750 mg, and more than 100 kg: 1000 mg abatacept) at day zero and switched back to SC abatacept (125 mg/week) at day 28. All patients were followed until day 168. Concomitant treatment with synthetic DMARDs (csDMARDs) was mandatory. The study was conducted in accordance with good clinical practice and the Declaration of Helsinki and was approved by an institutional review committee at each participating center (Ethikkommission des Kantons St. Gallen, Ethikkommission Nordwest- und Zentralschweiz, kantonalen Ethikkommission Bern, Ethikkommission des Kantons Waadt, and Swissmedic). All participating patients provided written informed consent (NCT1846975).

### Participants

Eligible patients were diagnosed with RA, as defined by the American College of Rheumatology/European League Against Rheumatism 2010 criteria [9], of more than 3 months’ duration as of the date of inclusion. Effective control of disease activity at baseline as defined by a DAS-28 (erythrocyte sedimentation rate, or ESR) of not more than 3.2 (LDA) was a prerequisite, and double-method contraception was mandatory during the study. Patients had to have been under continuous SC abatacept treatment for at least 3 months before the start of the study and remained under continuous synthetical DMARD therapy throughout the study. Oral corticosteroids (≤10 mg/day prednisone equivalent) were permitted with stable dosing during the month before baseline and throughout the study period. Patients who had failed more than two biological DMARDs other than abatacept or who had active uncontrolled disease, chronic infection, or a history of cancer in the last 5 years, other than non-melanoma skin cell cancers cured by local resection or carcinoma *in situ*, were excluded from the study.

### Assessments

The primary efficacy endpoint was the proportion (percentage) of subjects still in LDA (or in remission) on day 28; the proportions of patients in LDA on days 84 and 168 (versus baseline, when the IV abatacept infusion was administered) were secondary endpoints. Change from baseline evaluation of DAS-28 (ESR) score, health assessment questionnaire disability index (HAQ-DI) [10], and percentage of patients remaining on therapy with SC abatacept on days 28, 84, and 168 were analyzed as secondary endpoints. Treatment-emergent AEs and severe AEs relating to the switch to IV abatacept and the return to weekly SC abatacept were analyzed on days 28 and 168, respectively. Whether IV abatacept or tumor necrosis factor (TNF) antagonist pre-exposition influenced the occurrence of AEs or the evolving disease activity was also analyzed.

### Statistical analyses

The data were exported from secuTrial (interActive Systems GmbH, Berlin, Germany) on December 19, 2014. The statistical analysis was a per protocol (PP) analysis for the efficacy endpoints and an intention-to-treat (ITT) analysis for safety.

There were no missing data for the primary endpoint. Thus, the PP analysis was equal to a complete case analysis. For the secondary endpoints, a complete case analysis was performed for continuous variables. The two patients who dropped out of the study were calculated as therapeutic failures for the analysis.

For quantitative variables, descriptive statistics were employed. The primary endpoint was the proportion of patients with DAS-28 of not more than 3.2. Corresponding 95 % confidence intervals (CIs) for binomial probabilities are also reported (Wilson method). IV abatacept pre-exposed versus non-pre-exposed patients as well as patients with TNF antagonist versus patients without TNF antagonist pre-exposure were compared with a Mann-Whitney *U* test for continuous variables and with a Fisher’s exact test for binary variables. The change from baseline for DAS-28 and the HAQ-DI throughout the study period were compared by using linear mixed models (with random intercept and slope) with baseline as a predictor and time point as a covariate. Statistical analyses were performed with Statistical Package for the Social Sciences (SPSS) and the R programming language (version 3.1.0, R CORE TEAM [2013]; R: A Language and Environment for Statistical Computing; R Foundation for Statistical Computing, Vienna, Austria; http://www.R-project.org). The package Hmisc (Harrell, Frank E. Jr., with contribution form Charles Dupont and many others [2013]; Hmisc: Harrell Miscellaneous; R package version 3.13-0; http://CRAN.R-project.org/package=Hmisc) was used to compute CIs for proportions, and the package lme4 (Bates, Douglas, Maechler, Martin, Bolker, Ben, Walker, Steven [2013]; lme4: Li near mixed-effects models using Eigen and S4; R package version 1.0-5; http://www.inside-r.org/packages/lme4/versions/1-0-5) was used to compute linear mixed models.

## Results

### Patient characteristics and disposition

In total, 52 patients were included in the study (ITT). Three patients did not fulfill one of the inclusion criteria (DAS-28 of not more than 3.2) but were included by the principal investigator’s decision. The reasons for not fulfilling all inclusion criteria were transient ESR elevation in one case and increased patient’s global assessment of disease activity in another two, all of which quickly normalized between visit 1 and 2 (i.e., within 4 weeks), and all three patients were in LDA during almost the complete follow-up. However, these patients were excluded from the PP analysis. Fifty of the 52 patients completed the 24-week study. Patient demographics and baseline characteristics were similar in both analyses (ITT and PP) and indicated low baseline disease activity and longstanding disease (Table 1). One patient dropped out on day 28 and one on day 84, the first because of a flare and the second on the patient’s decision despite constant LDA. Both patients were counted as therapeutic failures for the analysis.

**Table 1:** Demographical data (at baseline)

	Intention to treat	Per protocol
Number, number	52	49
Gender, female/male	31/21	29/20
Age, years	59.1 ± 10.8	59.3 ± 11.0
Body mass index, kg/cm	27.8 ± 5.3	27.8 ± 5.4
Disease duration, years	8.7 ± 8.2	8.3 ± 7.7
HAQ-DI, baseline	0.55 ± 0.66	0.58 ± 0.67
Tender joint count 68, mean ± SD	1.95 ± 2.97	2.11 ± 2.95
Swollen joint count 68, mean ± SD	0.55 ± 1.29	0.60 ± 1.30
Erythrocyte sedimentation rate, mm/h	11.37 ± 6.00	11.38 ± 7.0
C-reactive protein, mg/l	3.49 ± 2.00	6.28 ± 3.0
Patient’s global assessment of disease activity (VAS 0–100)	16.97 ± 13.26	15.81 ± 17.90
Patient’s global assessment of pain (VAS 0–100)	19.62 ± 16.61	18.05 ± 17.58
Physician’s global assessment of disease activity (VAS 0–100)	10.98 ± 9.28	14.33 ± 7.45
DAS-28	1.73 ± 0.72	1.82 ± 0.80
Rheumatoid factor-positive^a^	62.0 %	63.8 %
ACPA-positive^a^	50.0 %	51.1 %
Erosive disease^a^	58.3 %	55.5 %
Pre-exposed to IV abatacept	38.5 %	34.6 %
Pre-exposed to TNF antagonists	60.1 %	53.4 %
Pre-exposed to other biologic agents	28.8 %	26.9 %
Concomitant therapy		
Leflunomide	n = 20	n = 19
Leflunomide + hydroxychloroquine	n = 1	n = 1
Methotrexate^b^	n = 18	n = 16
Hydroxychloroquine	n = 5	n = 5
Sulfasalazine	n = 4	n = 4
Sulfasalazine + hydroxychloroquine	n = 3	n = 3
Prednisolone dose or equivalent, mg/d	2.66 ± 3.25	2.82 ± 3.28

^a^Measured on patients with data assessable

^b^Methotrexate was always applied in subcutaneous formulation

*HAQ-DI* Health assessment questionnaire disability Index, *SD* standard deviation, *ACPA* anti-citrullinated peptide antibodies, *VAS* visual analog scale, *DAS-28* disease activity score in 28 joints

**Table 2:** Adverse events: weeks 0 through 24

System	Number	Description
Musculoskeletal	31	Arthritis/arthralgia (15), back pain (4), unspecific pain (3) osteopenia/osteoporosis (3), morning stiffness (1), tendovaginosis stenosans (1), plantar fascia pain (1), worsening fibromyalgia (1), tendinitis calcarea (1)
Infection	25	Upper airway infection (18), herpes labialis (2), fungi infection (2), fever (3)
Gastrointestinal	13	Vomiting (4), diarrhea (3), flatulence (1), diabetes mellitus (1), abdominal cramps (1), esophageal reflux (1), aphthous ulcers (1), lip blisters (no herpes proven, 1)
Neuro-psychological	14	Patient falling (4), restless legs (1), neuropathy (1), reduced leg strength (1), fatigue (1), anxiety (1), dysesthesia (1), depression (1), hyperventilation (1), involuntary movements (1), dizziness (1)
Other	7	Leg edema (2), polyp on vocal cord (2, same patient), hypokalemia (1), suspected claudicatio (1), vitamin D deficiency (1),
Dermatological	6	Exanthema (2), hematoma (2), hair loss (1), red eye (1)

### Clinical efficacy

**Fig. 1:**
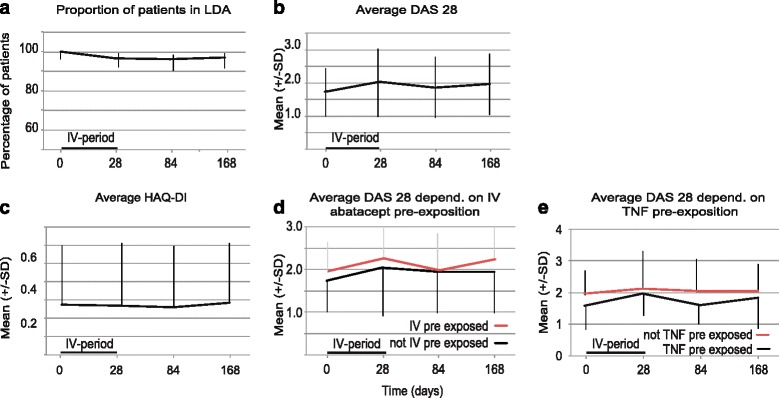
Disease activity per protocol. **a** The proportion of patients in low disease activity (LDA) was depicted with the 95 % confidence interval (CI) as an error bar. The interval of the intravenous (IV) abatacept between days 0 and 28 is marked with a bar. Before and after this time frame, the patient was treated with subcutaneous (SC) abatacept. **b** The mean disease activity score in 28 joints (DAS-28) was depicted for all patients with the standard deviation (SD) as error bars. **c** The mean health assessment questionnaire disability index (HAQ-DI) was depicted for all patients with the SD as error bars. **d** The mean DAS-28 was depicted for patients pre-exposed to tumor necrosis factor (TNF) antagonists (*black*) and not pre-exposed (*red*) separately with the SD as error bars. **e** The mean DAS-28 was depicted for patients pre-exposed to IV abatacept (*black*) and not pre-exposed (*red*) separately with the SD as error bars

In total, 46 out of 49 patients (PP analysis) were still in LDA after 28 days (ITT: 49/52). Thus, the proportion of patients with DAS-28 of not more than 3.2 as the primary endpoint was 93.3 % (95 % CI 83.5–97.9; Fig. 1a). Two out of the three patients with a DAS-28 of more than 3.2 on day 28 were back to LDA at the end of the study (i.e., on day 168) without any additional intervention. The average DAS-28 values were 1.73 (standard deviation (SD) ± 0.72) at baseline, 2.03 (SD ± 1.03) on day 28 and 1.96 (SD ± 0.97) at the end of the study (Fig. 1b). Pre-exposition to IV abatacept (Fig. 1c) or TNF antagonists (Fig. 1d) did not significantly influence the average DAS-28 or the proportion of patients retaining LDA.

### Patient-reported outcome

The average HAQ-DI values were 0.55 (SD ± 0.66) at baseline, 0.54 (SD ± 0.68) at day 28, and 0.56 (SD ± 0.66) at the end of the study (Fig. 1e, PP analysis). Pre-exposition to IV abatacept or TNF antagonists did not significantly influence the average HAQ-DI 28 (data not shown). The linear mixed model did not indicate any significant effect of time on HAQ-DI (estimate −5.28 × 10^−5^, 95 % CI −7.82 × 10^−4^–8.87 × 10^−4^).

### Safety

Out of 52 patients (ITT), 39 patients had a total of 96 AEs with a maximum of seven AEs per patient; 67 % of the AEs were judged by the treating physician as not to be related, 5.2 % as unlikely, 25 % as possibly, 1 % as probably, and 1 % as definitely related to IV abatacept as the study drug. All AEs, which were possibly, probably, or definitely related to study drug were of mild intensity. No AEs of special interest (cancer, overdose, transmission of an infectious agent via the medical product, potential drug-induced liver injury, allergic reactions, or blood dyscryasis) were reported. Twenty percent of the AEs occurred during the first 28 days after IV infusion of abatacept. Pre-exposure to IV abatacept or TNF antagonists did not influence the frequency of AEs.

In total, four serious AEs were described during the study: nodule on the vocal cord, subcutaneous leg edema, collapse at home, and depression. All serious AEs resulted in a hospitalization and were resolved during this hospitalization (Table 2).

## Discussion

This prospective, open-label, 24-week study of abatacept demonstrated for the first time in a controlled study setting that switching from weekly SC to IV abatacept and back is effective and safe and may be used to bridge 4 weeks in RA patients in DAS28-defined LDA or in remission.

Reggia et al. [11] have described a loss of efficacy after switching from IV to SC abatacept. A similar observation could not be made in our study, where the vast majority of patients remained in LDA not only at the primary endpoint on day 28 but also 6 months after switching back from IV to SC abatacept. There may be several reasons explaining this discrepancy. In our study, all patients had been pre-exposed to SC abatacept and were in stable LDA or in remission already at the beginning of the study, whereas in the setting of Reggia et al., patients were automatically switched from continuous IV abatacept to the SC formulation. It has to be kept in mind that the study by Reggia et al., in contrast to ours, was not a prospective study but an observational retrospective study and that minimal increases in DAS-28 could already have met their internal definition of a flare. This definition was not provided in their article. Similar maintenance of the good status LDA or remission after switching from IV to SC abatacept was found by others in the context of clinical trials [8] or in a case series [12]. This lack of a difference in clinical effectiveness in a drug with two pharmaceutical formulations is to be expected, in particular with regard to the previously reported non-inferiority in efficacy and safety for IV and SC abatacept in the ACQUIRE (Abatacept Comparison of Subcutaneous versus Intravenous in Inadequate Responders to Methotrexate) study [6].

### Importance of practical issues

The treatment of rheumatological patients while aiming to treat to target and achieving LDA or remission should also consider practical aspects of the patients’ daily lives. Patients, as is their natural right, try to lead a life which is as normal as possible despite their disease. Even though LDA or remission is the basis for this, it has to be considered that reaching an artificial target such as a calculated pre-defined DAS-28 level should not intervene with the patients’ individual demands and perception of life. Administration of only one IV dose may help patients on abatacept to cover even longer periods of absence for vacations or other reasons (e.g., business trips).

### Safety

The safety profile was similar to that observed in previous abatacept studies [2, 3, 6, 13]. Importantly, the rate of AEs did not increase within the first 28 days after IV application. The time interval permitted between the last SC dose and the IV abatacept application during the study ranged from 4 to 11 days. Whether shorter intervals between the SC and IV application of abatacept may increase the AE rate remains open but does not seem likely.

### Limitations

The extent of abatacept’s contribution to the maintenance of LDA or remission in the patients of our study with longstanding disease and concomitant DMARD therapy in all patients is uncertain. Only a randomized placebo controlled study might have shown a difference between the flare rate after a placebo and a verum IV abatacept application.

Even though such as study has not been conducted so far, there are some interesting observations in others. In the AVERT (Assessing Very Early Rheumatoid arthritis Treatment) study, early arthritis patients naïve to MTX were treated with SC abatacept and MTX [14]. All therapies (abatacept, MTX, and glucocorticosteroids) were stopped in patients reaching LDA. Within 4 weeks, the rate of patients in remission dropped from 61.3 % to about 51 % (-16.8 % of the patients in remission). In the ALLOW () trial, a temporary withdrawal of SC abatacept but not of MTX resulted in a slight increase of DAS-28 after 3 months, followed by complete recovery after reintroduction of abatacept [3].

All patients on MTX were treated with subcutaneously which is supplied by the manufacturer in prefilled syringes and, contrary to SC abatacept, is stable at room temperature for up to 2 years after manufacturing. Fragility could still pose the same problems as for SC abatacept. Therefore, replacing SC with oral MTX could be an option which was not investigated in our study.

The AE rate in total appears to be higher as compared with other studies with SC abatacept. However, musculoskeletal problems, including arthralgia and arthritis (15.6 % of all AEs), counted as AEs. Furthermore, selection of few sites with known high-quality standards may have led to a higher frequency of documented AEs. Importantly, no AEs of special interest such as defined for this study (cancer, overdose, transmission of an infectious agent via the medicinal product, potential drug-induced liver injury, allergic reactions, and blood dyscrasias) were documented, and all serious AEs were resolved during inpatient treatment.

Whether radiographic data would have been important for this analysis is a disputable issue. In our opinion, the follow-up was too short and the study would have required many more patients to be able to show adequate changes during radiographic progression.

## Conclusions

This study demonstrated the effectiveness and safety of switching from SC to IV abatacept before a vacation or business trip and then continuing with SC abatacept 4 weeks later. It is the first study to demonstrate this in a prospective manner. In our view, it emphasizes the need for both ways of abatacept administration. Whether this may also be true for other biologic agents available in both formulations remains open without further studies.

## Abbreviations

AE: adverse event
CI: confidence interval
DAS-28: disease activity score in 28 joints
DMARD: disease-modifying anti-rheumatic drug
ESR: erythrocyte sedimentation rate
HAQ-DI: health assessment questionnaire disability index
ITT: intention to treat
IV: intravenous
LDA: low disease activity
MTX: methotrexate
PP: per protocol
RA: rheumatoid arthritis
SC: subcutaneous
SD: standard deviation
TNF: tumor necrosis factor
